# Efficacy of combined gonadotropin-releasing hormone analogue and growth hormone therapy in girls with central precocious puberty: a systematic review and meta-analysis

**DOI:** 10.3389/fendo.2025.1662808

**Published:** 2025-10-07

**Authors:** Sufeng Jin, Yanhong Sun, Zhouyue Zhu, Haitao Yu

**Affiliations:** ^1^ Department of Clinical Laboratory, The Children’s Hospital, School of Medicine, Zhejiang University, National Clinical Research Center for Child Health, Hangzhou, China; ^2^ Zhejiang University-University of Edinburgh Institute (ZJU-UoE Institute), University School of Medicine, International Campus, Zhejiang University, Haining, Zhejiang, China; ^3^ Department of Clinical Laboratory, Sir Run Run Shaw Hospital, School of Medicine, Zhejiang University, Hangzhou, Zhejiang, China

**Keywords:** central precocious puberty, gonadotropin-releasing hormone analogue, growth hormone, final height, height gain, meta-analysis

## Abstract

**Background:**

Central precocious puberty (CPP) in girls is characterized by premature activation of the hypothalamic-pituitary-gonadal axis, often leading to early epiphyseal closure and compromised adult height. While gonadotropin-releasing hormone analogues (GnRHa) are the standard therapy to suppress puberty and preserve height potential, the benefit of adding growth hormone (GH) to improve height outcomes remains unclear. This work aims to evaluate the efficacy of combined GnRHa and GH therapy compared to GnRHa monotherapy in improving growth outcomes in girls with CPP.

**Methods:**

A systematic search of PubMed, Embase, Web of Science, and Cochrane Library was conducted up to May 2025. Eligible studies comparing GnRHa + GH combination therapy to GnRHa monotherapy in girls with CPP were included. Primary outcomes included final height and final height minus target height (FH–TH). Secondary outcomes included predicted adult height (PAH), height gain, height change during treatment, growth velocity, and bone maturation (ΔBA/ΔCA). Pooled weighted mean differences (WMDs) and 95% confidence intervals (CIs) were calculated using fixed- or random-effects models based on heterogeneity. Subgroup analyses were conducted by study design.

**Results:**

Nine studies were included. Combination therapy significantly improved FH–TH (WMD = 1.01 cm, 95% CI: 0.28 to 1.73; P = 0.006), PAH (WMD = 4.27 cm, 95% CI: 3.47 to 5.08; P < 0.0001), height gain (WMD = 3.45 cm, 95% CI: 1.73 to 5.17; P < 0.0001), height change during treatment (WMD = 3.31 cm, 95% CI: 1.76 to 4.86; P < 0.0001), and growth velocity (WMD = 1.40 cm/year, 95% CI: 0.90 to 1.91; P < 0.0001), with no significant effect on bone maturation (ΔBA/ΔCA) (WMD = 0.01, 95% CI: –0.05 to 0.07; P = 0.77). No significant improvement in final height was observed (WMD = 0.14 cm, 95% CI: –1.66 to 1.94; P = 0.88).

**Conclusion:**

GH supplementation during GnRHa treatment in girls with CPP enhances short-term growth outcomes without accelerating bone age but does not consistently improve final adult height. Combination therapy may be considered for selected patients with poor growth prognosis; however, further high-quality randomized trials are needed to refine patient selection and optimize treatment strategies.

## Introduction

Central precocious puberty (CPP) is a condition marked by the early activation of the hypothalamic–pituitary–gonadal (HPG) axis, resulting in the premature development of secondary sexual characteristics, accelerated bone age advancement, and a potential compromise in adult height among affected girls (1, 2). The standard first-line treatment for CPP is gonadotropin-releasing hormone analogue (GnRHa) therapy, which effectively halts further pubertal progression by suppressing pituitary gonadotropin secretion (3, 4). Although GnRHa has been shown to delay skeletal maturation and stabilize growth patterns, its effect on significantly increasing final adult height remains inconsistent, particularly in girls with delayed diagnosis, older chronological age, or already advanced bone age at initiation (5–7).

To address these limitations, the addition of growth hormone (GH) to GnRHa has been explored as a strategy to augment height potential during treatment. GH promotes longitudinal bone growth through stimulation of insulin-like growth factor-1 (IGF-1) and can potentially offset the deceleration in growth velocity commonly observed during GnRHa-induced pubertal suppression (8, 9). Several clinical studies and observational cohorts have investigated this combination approach, suggesting variable degrees of benefit in terms of final height, predicted adult height (PAH), height gain, and growth velocity (10–12). However, the interpretation of these results remains complex due to heterogeneity in baseline characteristics, treatment regimens, and outcome definitions.

Some reports indicate that combined GH and GnRHa therapy may be particularly advantageous in specific subgroups, such as those with initially low PAH or poor growth velocity after GnRHa initiation (13, 14). Conversely, other investigations have failed to demonstrate a meaningful difference between combination and monotherapy, raising concerns about overtreatment, increased cost, and potential side effects (15, 16). The existing literature also varies in methodological rigor, with randomized controlled trials (RCTs), prospective studies, and retrospective analyses yielding mixed conclusions.

Given the ongoing controversy and the clinical imperative to optimize growth outcomes in CPP, a systematic and quantitative synthesis of the available evidence is essential. A rigorous meta-analysis can clarify whether adjunctive GH confers additional benefit over standard GnRHa treatment, guide therapeutic decision-making in pediatric endocrinology, and inform future research priorities focused on individualized treatment strategies for children with CPP.

## Methods

### Search strategy

A comprehensive and systematic literature search was conducted across four major electronic databases: PubMed, Embase, Web of Science, and the Cochrane Library, covering the period from database inception to May 2025. The search strategy integrated both MeSH and free-text keywords to ensure broad coverage of studies related to CPP and its treatments. Key terms and their variants included: “central precocious puberty” or “CPP”; “gonadotropin-releasing hormone analogue” or “GnRHa”; “growth hormone” or “GH”; and outcome-related terms such as “final height,” “predicted adult height,” “height gain,” “growth velocity,” and “bone maturation.” The search was restricted to studies involving human subjects and published in English. Additionally, the reference lists of all eligible articles and pertinent reviews were manually screened to identify any potentially relevant studies that may have been missed during the initial database search.

### Eligibility criteria

Studies were included if they met the following criteria: the population comprised girls diagnosed with CPP, based on standard clinical and biochemical markers such as early onset of secondary sexual characteristics, advanced bone age, and a pubertal gonadotropin response to GnRH stimulation. The intervention involved combination therapy with GnRHa and GH, while the comparator group received GnRHa monotherapy. Eligible studies reported at least one of the following outcomes: final height, final height minus target height (FH–TH), PAH, height gain (defined as the difference between ffinal height and baseline PAH), height change during treatment, growth velocity (cm/year), or bone maturation (ΔBA/ΔCA) measured as the ratio of change in bone age to chronological age. Only clinical trials and retrospective studies with a comparative design were considered. Exclusion criteria encompassed studies involving patients with organic causes of precocious puberty (e.g., brain tumors, hypothalamic hamartomas), the use of other concurrent growth-affecting treatments (e.g., aromatase inhibitors), lack of a comparison group, insufficient quantitative data to compute effect sizes (means, standard deviations, or confidence intervals), and publication types such as reviews, case reports, letters, conference abstracts without full data, or duplicate reports.

### Study selection process

All records identified through the literature search were imported into EndNote X9, and duplicates were removed. Two independent reviewers screened the titles and abstracts for relevance. Full-text articles were retrieved for those that met the inclusion criteria or had unclear eligibility based on the abstract. Disagreements were resolved through discussion or consultation with a third reviewer.

### Data extraction

Two reviewers independently extracted data from each eligible study using a predesigned standardized data extraction form. Extracted information included the first author’s name, year of publication, country of origin, study design (RCT or observational study), sample size of each group, mean age at treatment initiation, duration of treatment and follow-up, and details of the intervention and comparator protocols, including drug type, dosage, and administration frequency. Outcome data collected included the mean and standard deviation (SD), or median and interquartile range (IQR), for FH–TH, PAH, height gain, height change during treatment, growth velocity, and bone maturation (ΔBA/ΔCA). Reported adverse events were also documented when available. For studies that presented medians and IQRs, the corresponding means and SDs were estimated using Wan’s method or Hozo’s formula, as appropriate.

### Quality assessment

The Cochrane Risk of Bias tool (RoB 2.0) was used to assess the quality of RCTs, examining domains such as randomization, allocation concealment, blinding, completeness of outcome data, and selective reporting. For non-randomized studies, the Newcastle–Ottawa Scale (NOS) was applied, which evaluates three domains: selection of study groups (4 points), comparability of groups (2 points), and ascertainment of outcomes (3 points). Studies scoring ≥6 points were considered moderate-to-high quality. All assessments were performed independently by two reviewers, with disagreements resolved by consensus.

### Statistical analysis

All statistical analyses were performed using RevMan (Review Manager) version 5.4. The effect size for continuous outcomes was expressed as weighted mean differences (WMDs) and 95% confidence intervals (CIs). Heterogeneity across studies was assessed using Cochran’s Q test and the I² statistic. A fixed-effects model was used when heterogeneity was low (I² ≤ 50%). In cases of substantial heterogeneity (I² > 50%), a random-effects model was applied to provide more conservative estimates. Subgroup analyses were predefined and performed to examine whether study design (clinical trials vs. retrospective studies) influenced the effect of combined therapy versus monotherapy. Publication bias was assessed visually using funnel plots.

## Results

### Study selection

**Figure 1:**
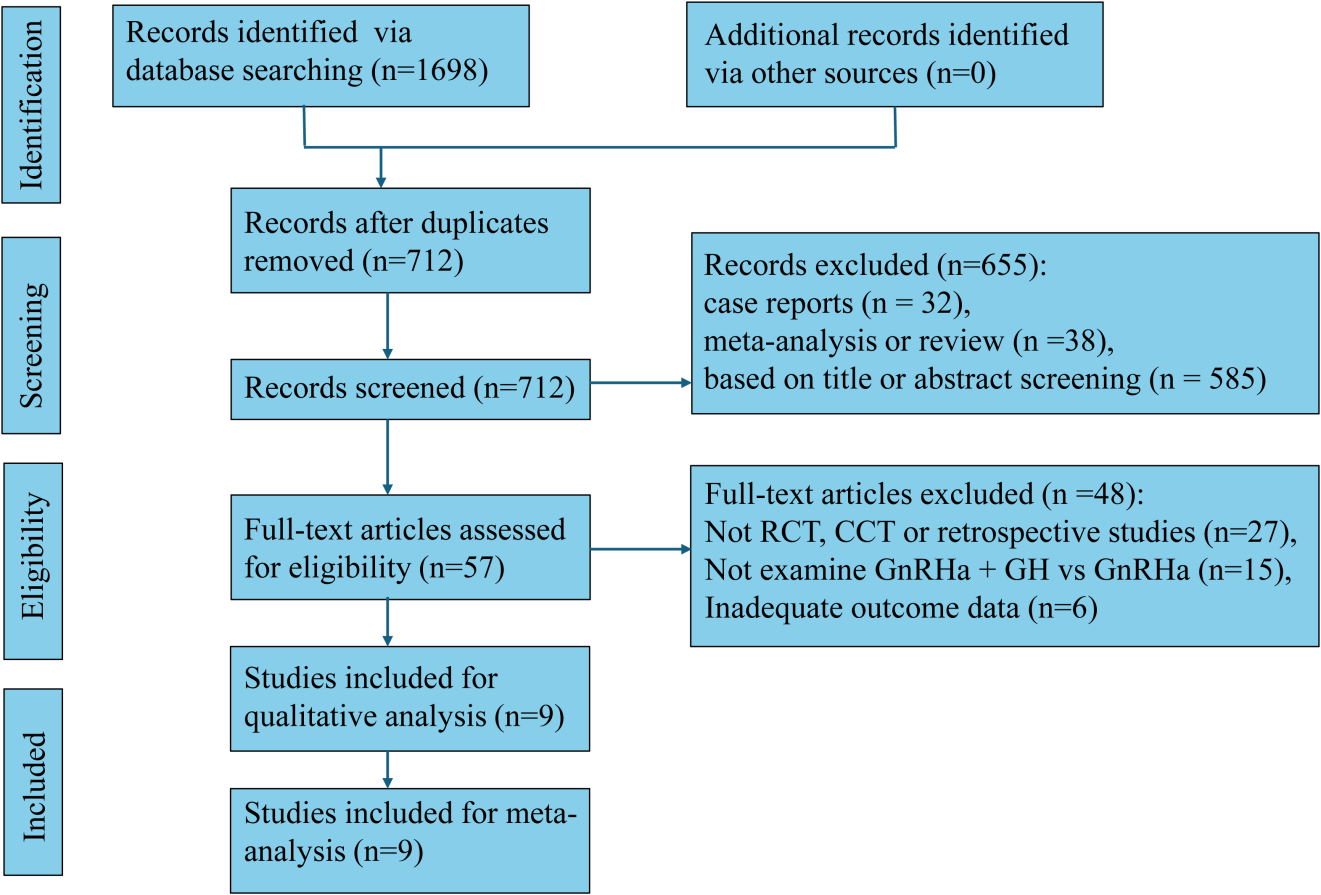
Flow diagram of meta-analysis.

A total of 1,698 records were identified through systematic database searching for studies comparing the efficacy of GnRHa combined with GH versus GnRHa alone in girls with CPP. No additional studies were found through other sources. After removing duplicates, 712 unique records were screened by title and abstract, resulting in the exclusion of 655 articles due to being case reports (n=32), meta-analyses or reviews (n=38), or irrelevant based on title or abstract (n=585). The remaining 57 articles were retrieved for full-text assessment, of which 48 were excluded for not being RCTs, case-control trials (CCTs), or retrospective studies (n=27), for not comparing the appropriate interventions (n=15), or for having inadequate outcome data (n=6). Ultimately, 9 studies fulfilled all inclusion criteria and were incorporated into both the qualitative synthesis and the final meta-analysis (**Figure 1**).

### Study characteristics

**Table 1** summarizes the key characteristics of the nine studies included in this meta-analysis, encompassing a total of 9 comparative cohorts evaluating the effects of GnRHa combined with GH versus GnRHa alone in girls with CPP. The studies were conducted in Korea (n=4), Italy (n=2), China (n=2), and the Netherlands (n=1), with publication years ranging from 2000 to 2024. Study designs included retrospective studies (n=5), CCT s(n=3), and one RCT. Sample sizes varied, with the number of participants in the GnRHa+GH group ranging from 10 to 46, and in the GnRHa-only group from 10 to 188. The mean chronological age at baseline and at the end of treatment ranged approximately from 7.8 to 9.6 years and 9.2 to 11.6 years, respectively, across studies. The GnRHa dosages used were generally consistent within the range of 60–150 µg/kg or fixed doses (e.g., 3.75 mg every 28 days), while GH dosages varied from 0.25 mg/kg/week to 4 IU/m²/day. The treatment duration with GnRHa prior to GH initiation ranged from several months up to 2 years, and the combined treatment duration ranged from approximately 1.9 to 3 years. Growth-related outcomes reported across studies included final height, FH-TH, height gain, growth velocity, bone maturation (ΔBA/ΔCA), PAH, and overall height changes. These comprehensive data provided the foundation for the subsequent quantitative synthesis and subgroup analyses. One stated that participants were randomly assigned to either the intervention or control group. There was no risk of bias from incomplete outcome data, as all patients completed the study with no loss to follow-up. For the remaining eight studies, the NOS scores ranged from 7 to 9, indicating high methodological quality across the cohort studies and supporting the reliability of the meta-analysis findings.

**Table 1:** Characteristics of the included studies.

Studies	Country	Study design	Number of patients		Chronological age at the start of treatment		Bone age at the start of treatment		GnRHa dose	GH dose	Growth variables reported	GnRHa treatment period before GH	GnRHa and GH treatment period
			GnRHa + GH	GnRHa	GnRHa + GH	GnRHa	GnRHa + GH	GnRHa					
Cho et al. (2023) (17)	Korea	Retrospective	22	188	8.34 ± 0.44	8.20 ± 0.62	10.51± 0.61	10.30 ± 0.77	1.87-3.75 mg every 28 days	0.6 IU/kg/wk (6 days weekly)	Final height, FH-TH, height gain, growth velocity, bone maturation (ΔBA/ΔCA), PAH changes	6.9 months	2.04 ± 0.90 years
Gyon et al. (2015) (11)	Korea	Retrospective	24	61	7.9 ± 0.7	8.2 ± 0.8	10.4 ± 1.3	10.5 ± 1.1	75-150 µg/kg	0.7 ± 0.1 IU/kg/week	Final height, FH-TH, height gain, growth velocity, bone maturation (ΔBA/ΔCA), height changes, PAH changes	NA	2.1 ± 1.1 years
Jung et al. (2014) (18)	Korea	Retrospective	23	59	8.8 ± 0.59	8.7 ± 0.78	10.5 ± 0.86	10.5 ± 0.86	75-150 µg/kg	0.25 mg/kg/week (6 days weekly)	Final height, FH-TH, height gain, growth velocity, bone maturation (ΔBA/ΔCA), height changes, PAH changes	NA	1.9 ± 0.99 years
Kim et al. (2019) (19)	Korea	Retrospective	31	135	7.81 ± 0.97	7.91 ± 0.77	9.77 ± 0.84	9.25 ± 1.10	60–90 µg/kg every 4 weeks	0.25 mg/kg/week	Final heigth, FH-TH, height gain, bone maturation (ΔBA/ΔCA), height changes, PAH changes	19.19 ± 19.48 months	39.23 ± 16.94 months
Mul et al. (2005) (20)	Netherlands	RCT	14	12	9.6 ± 0.9	9.6 ± 0.9	11.6 ± 0.8	10.7 ± 1.1	3.75 mg/28 days	4 IU/m^2^/day	Final heigjt, height gain, growth velocity, bone maturation (ΔBA/ΔCA), height changes, PAH changes	NA	3 years
Pucarelli et al. (2000) (12)	Italy	CCT	10	10	7.9 ± 0.6	7.6 ± 0.2	10.6 ± 0.4	10.4 ± 0.3	100 µg/kg/21 days	0.3 mg/kg/week (6 days weekly)	Final height, FH-TH, height gain, bone maturation (ΔBA/ΔCA), PAH changes	2 years	3.07 ± 1.33 years
Pucarelli et al. (2003) (17)	Italy	CCT	17	18	8.3 ± 1.6	7.9 ± 0.8	11.0 ± 1.4	10.7 ± 1.2	101 µg/kg/21 days	0.3 mg/kg/week (6 days weekly)	Final height, FH-TH, height gain, bone maturation (ΔBA/ΔCA), PAH changes	1.6 years	3 years
Shi et al. (2024) (21)	China	Retrospective	46	34	8.73 ± 0.94	8.02 ± 0.83	10.47 ± 1.01	9.24 ± 1.07	3.75 mg/4 weeks	0.05-0.066 mg/kg/day	Growth velocity, height changes, PAH changes	NA	≥30 months
Wang et al. (2014) (22)	China	CCT	31	49	9.2 ± 0.7	8.9 ± 0.6	11.2 ± 0.53	11.0 ± 0.5	100 µg/kg	0.12-0.15 IU/kg/day	Final height, FH-TH, height gain, growth velocity, bone maturation (ΔBA/ΔCA), height changes, PAH changes	25.3 ± 6.9 months	12.9 ± 7.0 months

Bone maturation (ΔBA/ΔCA), defined as change in bone age divided by change in chronological age progression; CCT, case–control trial; FH-TH, final height minus target height; GH, growth hormone; GnRHa, gonadotropin-releasing hormone analogue; PAH changes, predict adult height changes; RCT, randomized controlled trial.

### Meta-analysis of FH–TH

**Figure 2:**
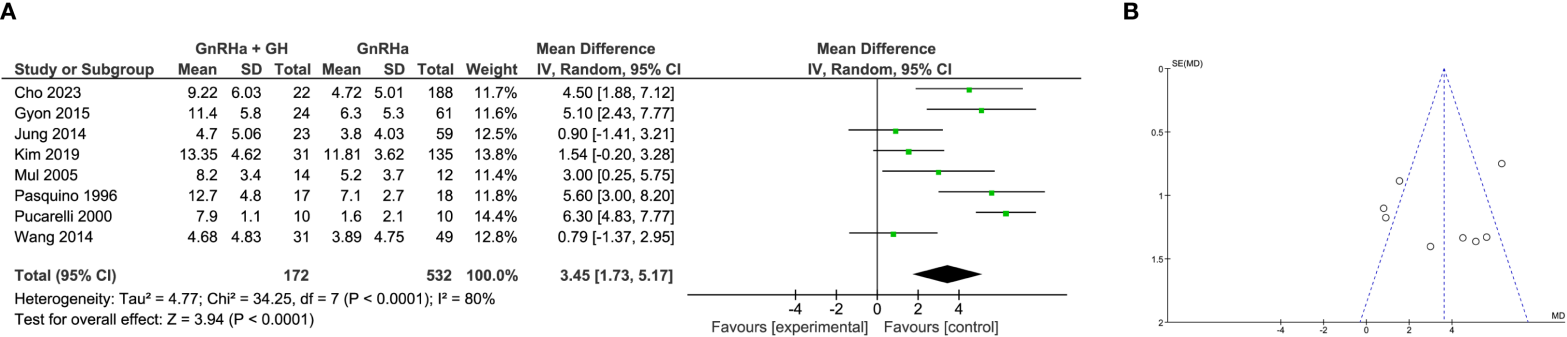
Meta-analysis of final height minus target height (FH-TH). **(A)** Forst plot of meta-analysis of FH-TH. **(B)** Funnel plot of studies reporting FH-TH.

To determine whether the addition of GH to GnRHa therapy yields a greater deviation of FH–TH, a total of 7 studies were included. Between-study heterogeneity was moderate (I² = 49%), and a fixed-effects model was employed for meta-analysis. The pooled WMD demonstrated that girls treated with GnRHa + GH had a significantly greater FH–TH compared to those receiving GnRHa alone (WMD = 1.01 cm, 95% CI: 0.28 to 1.73; P = 0.006; **Figure 2A**), indicating an improvement in height outcome beyond genetic expectation.

To investigate the potential influence of study design on treatment effect, subgroup analysis was conducted. In clinical trials, the benefit of combination therapy was more pronounced, with a statistically significant increase in FH–TH compared to monotherapy (WMD = 2.35 cm, 95% CI: 1.06 to 3.65; P = 0.0006; **Supplementary Figure S1**). Conversely, retrospective studies did not demonstrate a statistically significant advantage (WMD = 0.39 cm, 95% CI: –0.48 to 1.27; P = 0.38), suggesting that study type may moderate treatment efficacy. Funnel plot analysis revealed a symmetrical distribution of included studies, indicating minimal publication bias for this outcome (**Figure 2B**).

### Meta-analysis of final height

**Figure 3:**
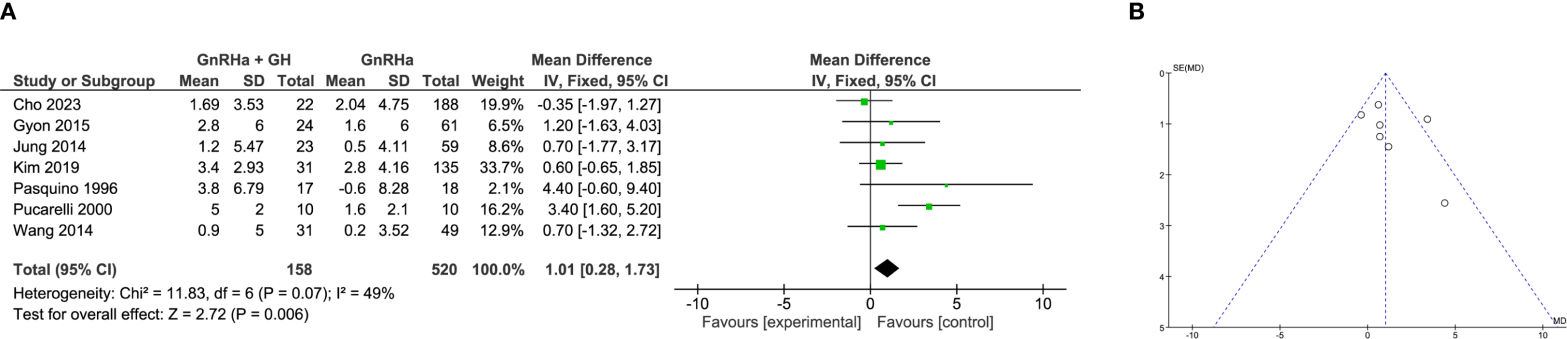
Meta-analysis of final height. **(A)** Forst plot of meta-analysis of final height. **(B)** Funnel plot of studies reporting final height.

A total of 8 studies evaluated the impact of GH addition on final adult height in CPP girls. Due to substantial heterogeneity among studies (I² = 81%), a random-effects model was applied. The pooled analysis indicated no significant improvement in final height for the combination therapy compared to GnRHa monotherapy (WMD = 0.14 cm, 95% CI: –1.66 to 1.94; P = 0.88; **Figure 3A**).

Subgroup analysis further clarified these findings. In clinical trials, a trend toward improved final height with GH co-treatment was observed but did not reach statistical significance (WMD = 2.14 cm, 95% CI: –0.14 to 4.43; P = 0.07). Interestingly, in retrospective studies, monotherapy with GnRHa appeared more effective, with a significantly greater final height compared to combination therapy (WMD = –1.75 cm, 95% CI: –2.65 to –0.85; P = 0.0001), raising questions about potential selection biases or unmeasured confounding in non-randomized data. Funnel plot analysis suggested no major asymmetry, supporting low risk of publication bias (**Figure 3B**).

### Meta-analysis of height gain

**Figure 4:**
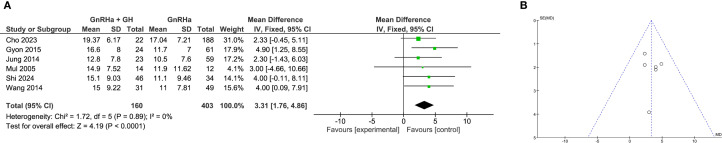
Meta-analysis of height gain. **(A)** Forst plot of meta-analysis of height gain. **(B)** Funnel plot of studies reporting height gain.

Height gain, defined as the difference between initial PAH and actual final height, was assessed in 8 studies. Despite substantial heterogeneity (I² = 80%), random-effects modeling revealed that combination therapy significantly improved height gain compared to GnRHa alone (WMD = 3.45 cm, 95% CI: 1.73 to 5.17; P < 0.0001; **Figure 4A**).

This positive effect was consistent across study designs. In clinical trials, the pooled WMD was 3.97 cm (95% CI: 1.24 to 6.70; P = 0.004), while in retrospective studies, the effect was slightly attenuated but remained significant (WMD = 2.86 cm, 95% CI: 0.88 to 4.84; P = 0.005; **Supplementary Figure S3**). These findings support the role of GH in augmenting linear growth beyond genetic expectations. Symmetrical funnel plots indicated a low likelihood of publication bias for this endpoint (**Figure 4B**).

### Meta-analysis of height change during treatment

**Figure 5:**
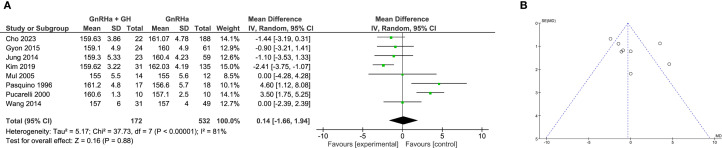
Meta-analysis of height changes during treatment. **(A)** Forst plot of meta-analysis of height changes during treatment. **(B)** Funnel plot of studies reporting height changes during treatment.

The effect of GH on height change during the treatment period was analyzed in 6 studies. Heterogeneity was negligible (I² = 0%), permitting the use of a fixed-effects model. Pooled results demonstrated that the GnRHa + GH group exhibited significantly greater height increment during treatment than the GnRHa alone group (WMD = 3.31 cm, 95% CI: 1.76 to 4.86; P < 0.0001; **Figure 5A**).

Subgroup analyses confirmed the robustness of this effect across study designs. In clinical trials, combination therapy led to a mean height increase of 3.79 cm over monotherapy (95% CI: 0.31 to 7.28; P = 0.03), whereas retrospective studies reported a comparable gain of 3.19 cm (95% CI: 1.47 to 4.92; P = 0.0003; **Supplementary Figure S4**). The results indicate that GH enhances growth trajectory even within the active treatment window. Funnel plots were symmetrical, further supporting the validity of the findings (**Figure 5B**).

### Meta-analysis of PAH

**Figure 6:**
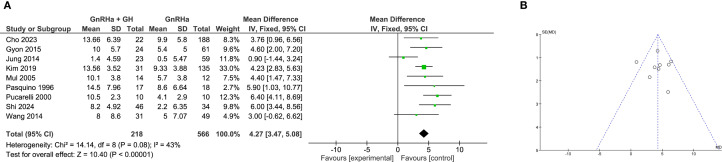
Meta-analysis of predicted adult height (PAH). **(A)** Forst plot of meta-analysis of PAH. **(B)** Funnel plot of studies reporting PAH.

A total of 9 studies examined changes in PAH. Between-study heterogeneity was moderate (I² = 43%), and a fixed-effects model was used. The pooled data showed that GH addition led to a significant increase in PAH compared to GnRHa alone (WMD = 4.27 cm, 95% CI: 3.47 to 5.08; P < 0.0001; **Figure 6A**).

Subgroup analysis again supported the efficacy of combination therapy. Clinical trials reported a greater increase in PAH (WMD = 5.19 cm, 95% CI: 3.66 to 6.72; P < 0.00001), while retrospective studies also confirmed a significant effect (WMD = 3.92 cm, 95% CI: 2.98 to 4.87; P < 0.00001; **Supplementary Figure S5**). These findings suggest that early prediction of adult height may be substantially improved with GH co-treatment. No significant publication bias was detected based on funnel plot analysis (**Figure 6B**).

### Meta-analysis of growth velocity

**Figure 7:**
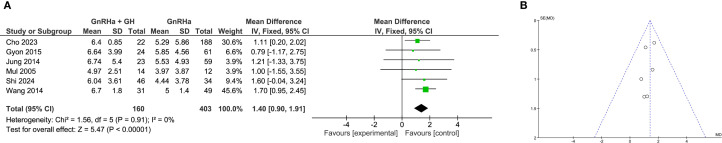
Meta-analysis of growth velocity. **(A)** Forst plot of meta-analysis of growth velocity. **(B)** Funnel plot of studies reporting growth velocity.

Growth velocity was reported in 6 studies. With no between-study heterogeneity (I² = 0%), a fixed-effects model was employed. The analysis revealed a significant improvement in growth rate among those receiving combination therapy (WMD = 1.40 cm/year, 95% CI: 0.90 to 1.91; P < 0.0001; **Figure 7A**).

Further breakdown showed that clinical trials demonstrated a stronger effect (WMD = 1.65 cm/year, 95% CI: 0.93 to 2.36; P < 0.00001), while retrospective studies also showed benefit (WMD = 1.17 cm/year, 95% CI: 0.46 to 1.88; P = 0.001; **Supplementary Figure S6**). These results indicate that GH supplementation meaningfully accelerates growth velocity during therapy. Funnel plot inspection did not suggest publication bias (**Figure 7B**).

### Meta-analysis of bone maturation (ΔBA/ΔCA)

**Figure 8:**
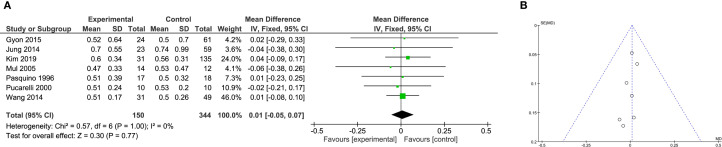
Meta-analysis of bone maturation. **(A)** Forst plot of meta-analysis of bone maturation. **(B)** Funnel plot of studies reporting bone maturation (ΔBA/ΔCA).

The impact of GH on bone maturation(ΔBA/ΔCA), measured as the ratio of bone age progression to chronological age progression, was analyzed in 7 studies. With no heterogeneity (I² = 0%), fixed-effects analysis indicated no significant difference between groups (WMD = 0.01, 95% CI: –0.05 to 0.07; P = 0.77; **Figure 8A**).

Subgroup analysis of both clinical trials (WMD = 0.00, 95% CI: –0.08 to 0.08; P = 0.98) and retrospective studies (WMD = 0.03, 95% CI: –0.09 to 0.14; P = 0.62; **Supplementary Figure S7**) consistently showed no effect. This suggests that while GH may promote linear growth, it does not accelerate skeletal maturation in the context of CPP treatment. Funnel plots were symmetric, implying low risk of bias (**Figure 8B**).

## Discussion

This meta-analysis provides a comprehensive synthesis of the available evidence comparing the efficacy of combined GnRHa and GH therapy versus GnRHa monotherapy in girls with idiopathicCPP. Our findings demonstrate that the addition of GH leads to significant improvements in several intermediate growth outcomes, including height gain,PAH, growth velocity, and height changes during treatment. These results align with prior studies suggesting that GH supplementation may help counteract the growth deceleration that commonly occurs during GnRHa-induced pubertal suppression (23–25).

However, despite these improvements in short-term and surrogate growth metrics, our meta-analysis found no statistically significant benefit of combination therapy on final adult height. This finding is consistent with several previous investigations, including randomized trials and longitudinal cohort studies, which reported limited or no added value of GH on final height when combined with GnRHa (26–28). In contrast, some studies have reported a positive final height effect, particularly in girls with severely compromised PAH at baseline or those who respond poorly to GnRHa alone (29, 30). These discrepancies may reflect variation in inclusion criteria, treatment initiation age, GH dosing, and duration of follow-up across studies.

Our findings regarding PAH improvement and enhanced height gain are in agreement with earlier literature suggesting that GH is particularly beneficial in cases with advanced bone age or low baseline growth velocity (31, 32). Studies have reported that PAH increased significantly in girls treated with combined therapy compared to those receiving GnRHa alone, especially in individuals with PAH below the normal range (33). Similarly, a meta-analysis by Liu et al. highlighted consistent improvements in PAH and height velocity among combination therapy recipients, although it also noted significant heterogeneity in final height outcomes (34). The current synthesis reaffirms these trends and further supports the value of PAH and growth velocity as sensitive indicators of treatment responsiveness.

Importantly, our analysis shows that GH supplementation does not significantly affect bone maturation (ΔBA/ΔCA), suggesting that the observed growth acceleration does not come at the cost of prematurely closing growth plates. This is in line with prior studies that have reported stable bone age progression rates during combined therapy (35, 36), reinforcing the safety of GH in this context regarding skeletal maturation. The maintenance of physiological bone development is a key consideration in evaluating the long-term effectiveness and safety of any intervention aimed at improving height outcomes in pediatric populations.

From a clinical standpoint, our findings suggest that GH co-treatment “may be considered for selected patients.” More specifically, the greatest potential benefit may be expected in girls with low PAH at baseline or in those demonstrating poor growth velocity during GnRHa monotherapy. However, the decision to initiate combination therapy must also weigh broader considerations, including the high financial cost of GH, the need for long-term injections, and the potential risk of overtreatment in children who may ultimately reach an acceptable adult height without adjunctive therapy.

This study has several strengths, including a rigorous methodological approach, pre-specified subgroup analyses, and a comprehensive assessment of multiple growth outcomes. This study has several limitations. First, although our meta-analysis included nine comparative studies, only one was a randomized controlled trial; the remainder were retrospective or case–control studies, which increases the risk of bias due to non-random treatment allocation and unmeasured confounding. Second, substantial heterogeneity was observed in some outcomes, particularly final height and height gain, likely reflecting differences in baseline characteristics, GH dosage, timing of initiation, treatment duration, and follow-up length across studies. Third, important clinical variables including age stratification (<10 vs. ≥10 years), whether GH was initiated after or concurrently with GnRHa, and whether patients received GH without GnRHa were not explicitly reported in the included studies, preventing further subgroup analyses. Fourth, factors such as sex, GH dose adjustment, follow-up IGF-1 levels, and adherence were inconsistently described, limiting our ability to evaluate their influence on treatment response. Finally, the included studies did not provide sufficient information to allow further analysis of high-risk subgroups, such as patients with severely compromised predicted adult height or poor growth velocity during GnRHa therapy. Future studies should stratify these subgroups to better define which patients are most likely to benefit from GH co-treatment.

## Conclusions

In summary, this meta-analysis shows that adding GH to GnRHa therapy in girls with CCP significantly improves intermediate growth outcomes, including height gain, PAH, growth velocity, and height change during treatment, without accelerating bone maturation. However, these short-term benefits did not translate into consistent improvements in final adult height, underscoring the need for cautious interpretation and individualized treatment decisions. Given the heterogeneity among studies and the limited number of high-quality randomized controlled trials, further research is needed to better define the patient subgroups most likely to benefit and to determine the optimal timing and duration of GH co-treatment. Future work should also consider cost-effectiveness and the potential risks of overtreatment to ensure that combined therapy is applied judiciously in clinical practice.

## Data Availability

The original contributions presented in the study are included in the article/**Supplementary Material**. Further inquiries can be directed to the corresponding author.

## Supplementary material

Supplementary Figure 1 Forest plot of subgroup analysis of final height minus target height (FH-TH).

Supplementary Figure 2 Forest plot of subgroup analysis of final height.

Supplementary Figure 3 Forest plot of subgroup analysis of height gain.

Supplementary Figure 4 Forest plot of subgroup analysis of height changes during treatment.

Supplementary Figure 5 Forest plot of subgroup analysis of predicted adult height (PAH).

Supplementary Figure 6 Forest plot of subgroup analysis of growth velocity.

Supplementary Figure 7 Forest plot of subgroup analysis of bone maturation (ΔBA/ΔCA).
