# Supplementary figures and images for: Efficacy of combined gonadotropin-releasing hormone analogue and growth hormone therapy in girls with central precocious puberty: a systematic review and meta-analysis
Source: Front Endocrinol (Lausanne). 2025 Oct 7;16:1662808. doi: 10.3389/fendo.2025.1662808 (PMC12537404; doi:10.3389/fendo.2025.1662808)

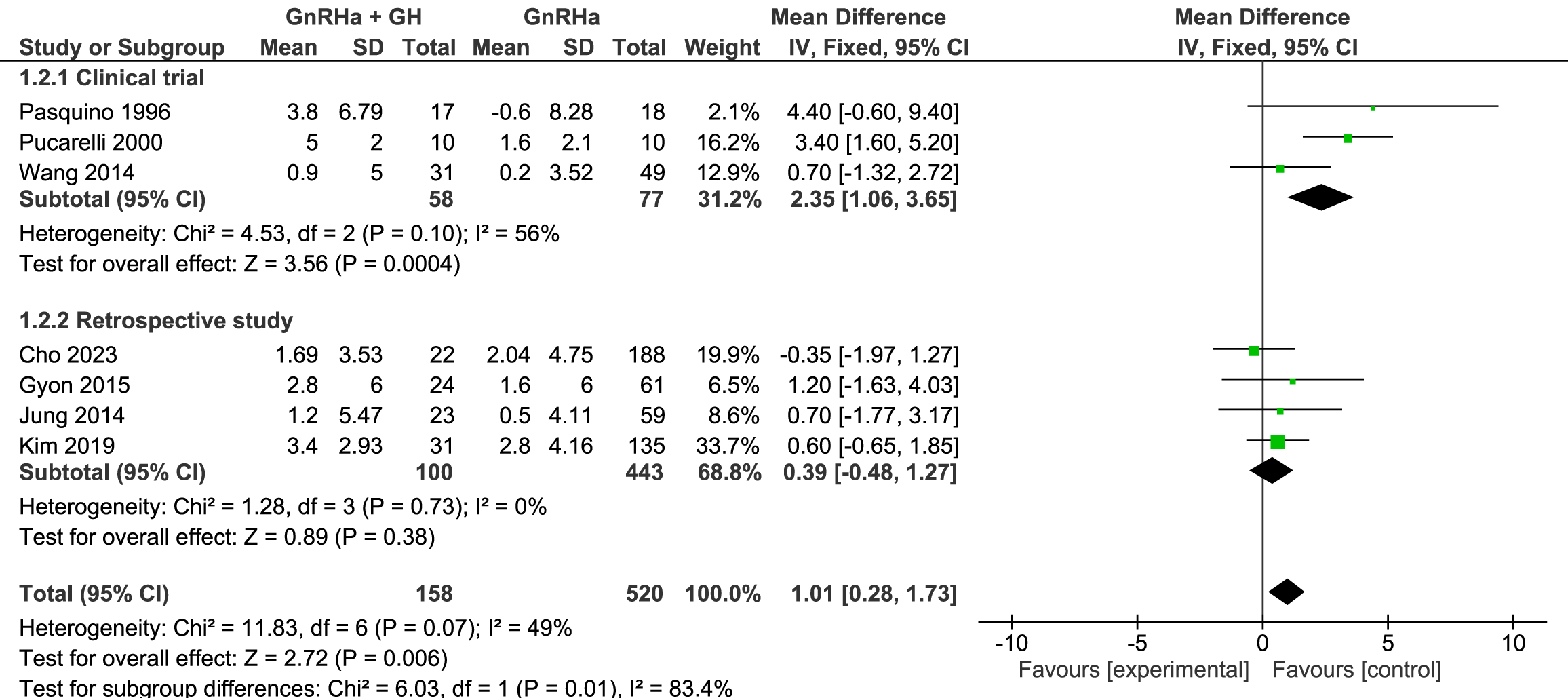

Supplement: Supplementary Figure 1 — Forest plot of subgroup analysis of final height minus target height (FH-TH). [file Image1.tif]

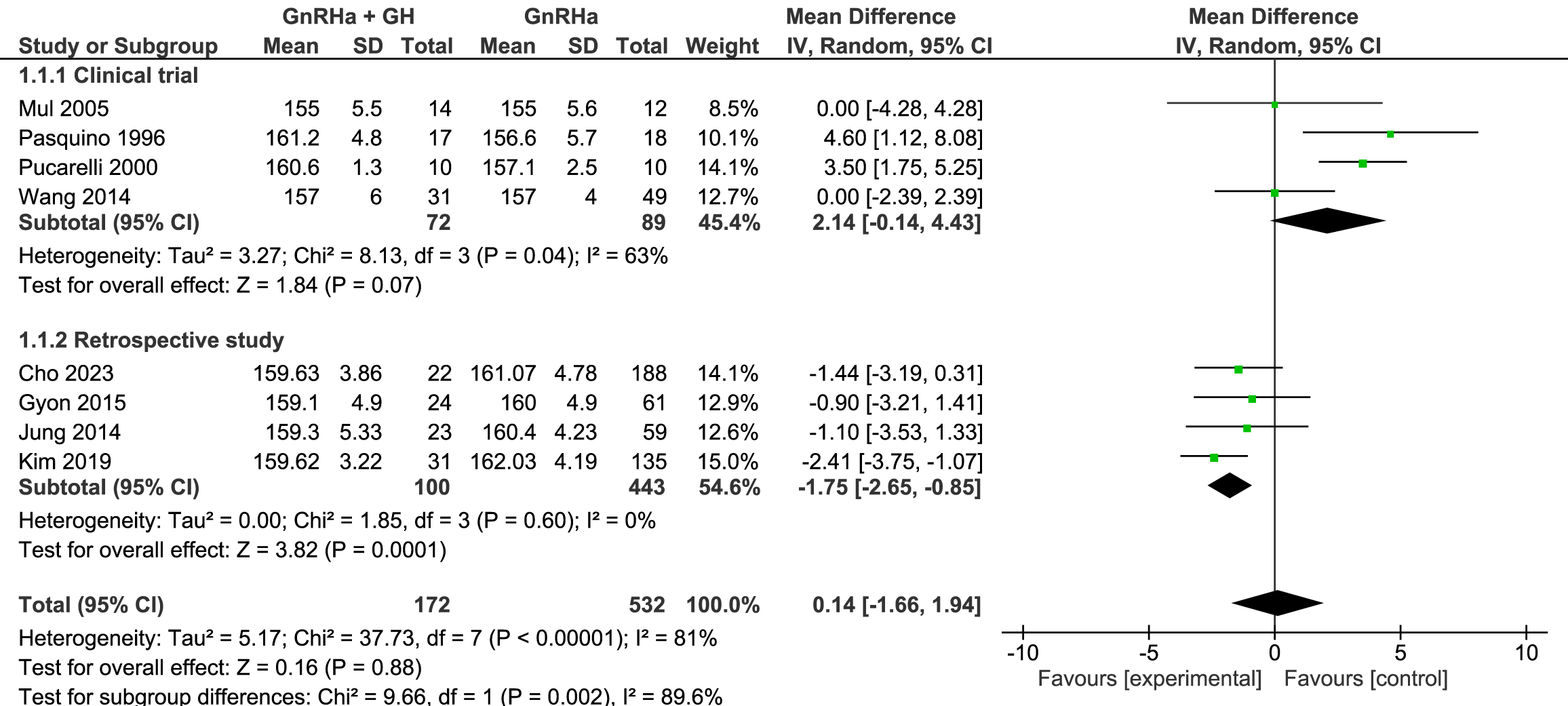

Supplement: Supplementary Figure 2 — Forest plot of subgroup analysis of final height. [file Image2.tif]

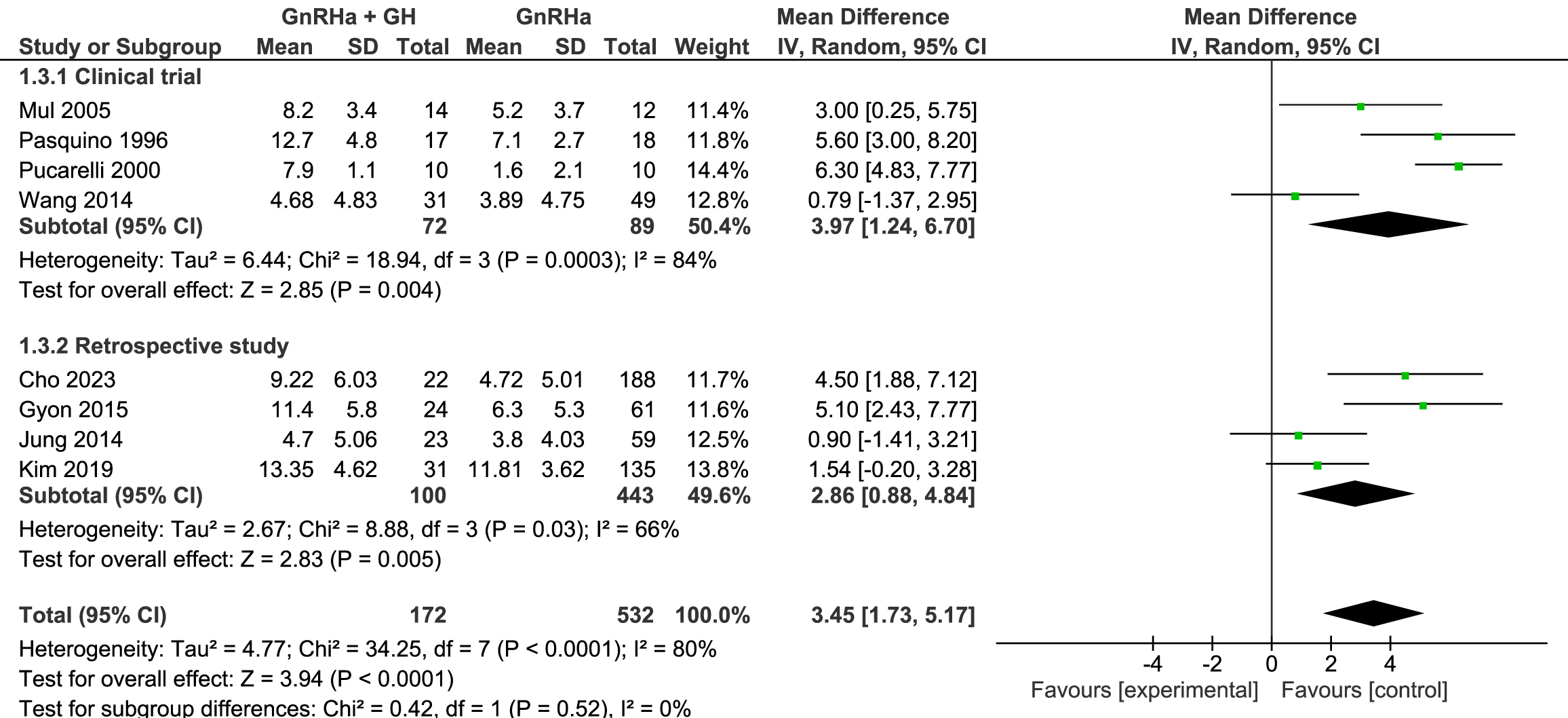

Supplement: Supplementary Figure 3 — Forest plot of subgroup analysis of height gain. [file Image3.tif]

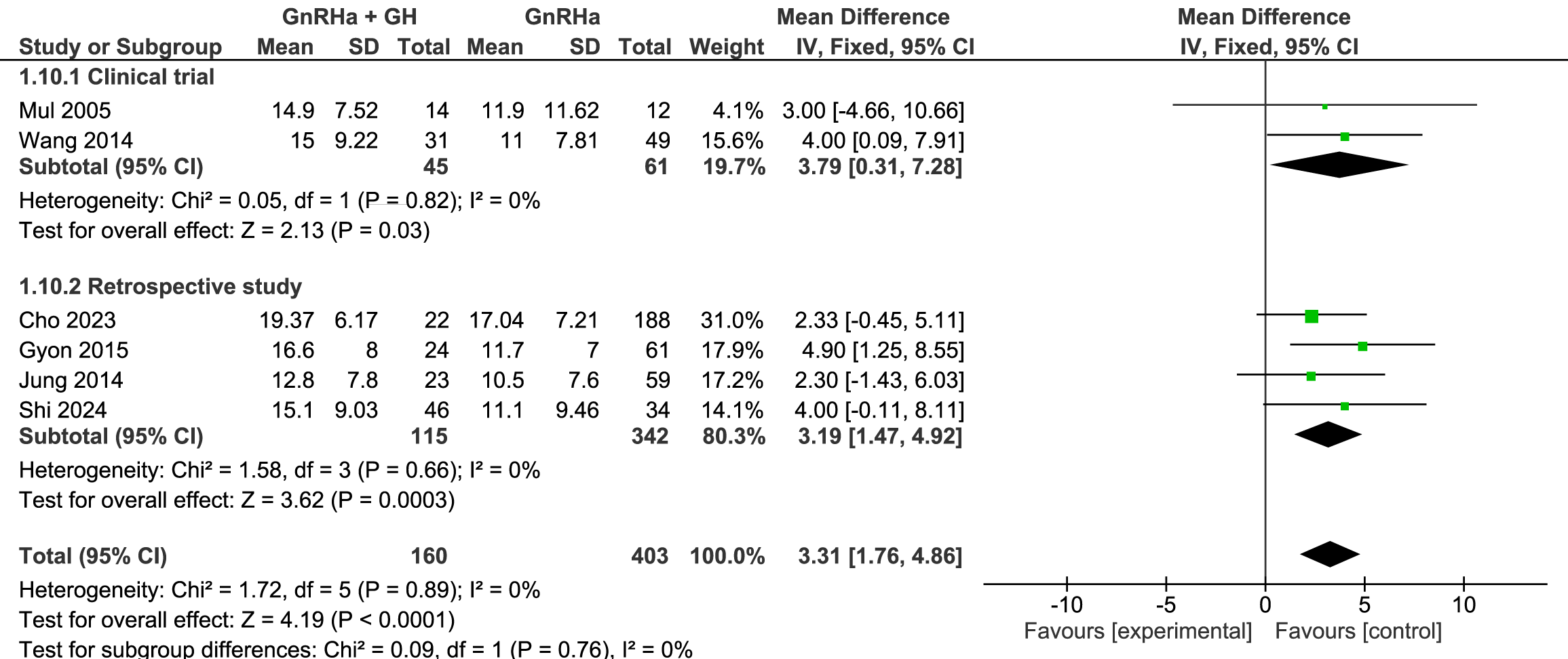

Supplement: Supplementary Figure 4 — Forest plot of subgroup analysis of height changes during treatment. [file Image4.tif]

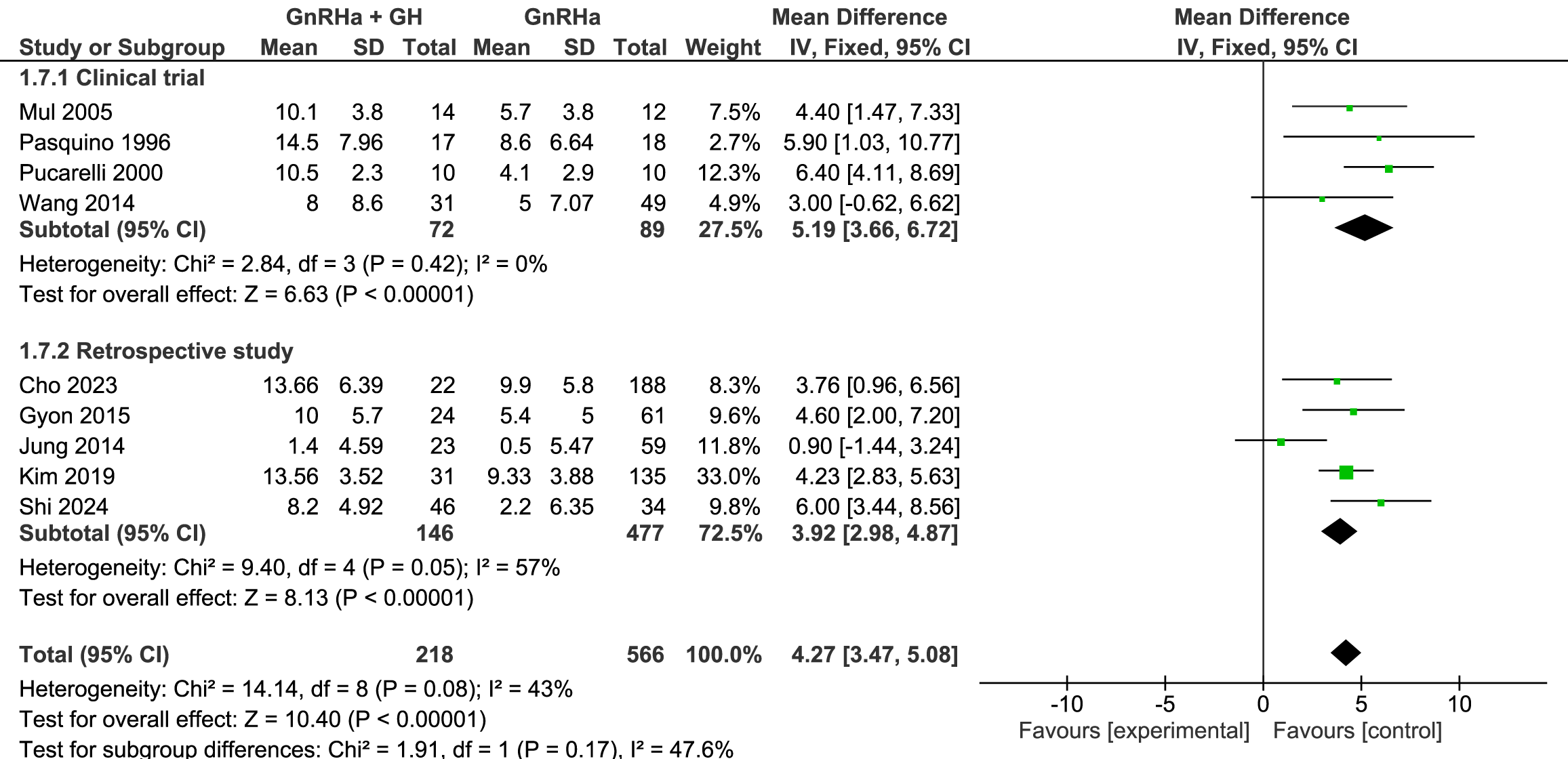

Supplement: Supplementary Figure 5 — Forest plot of subgroup analysis of predicted adult height (PAH). [file Image5.tif]

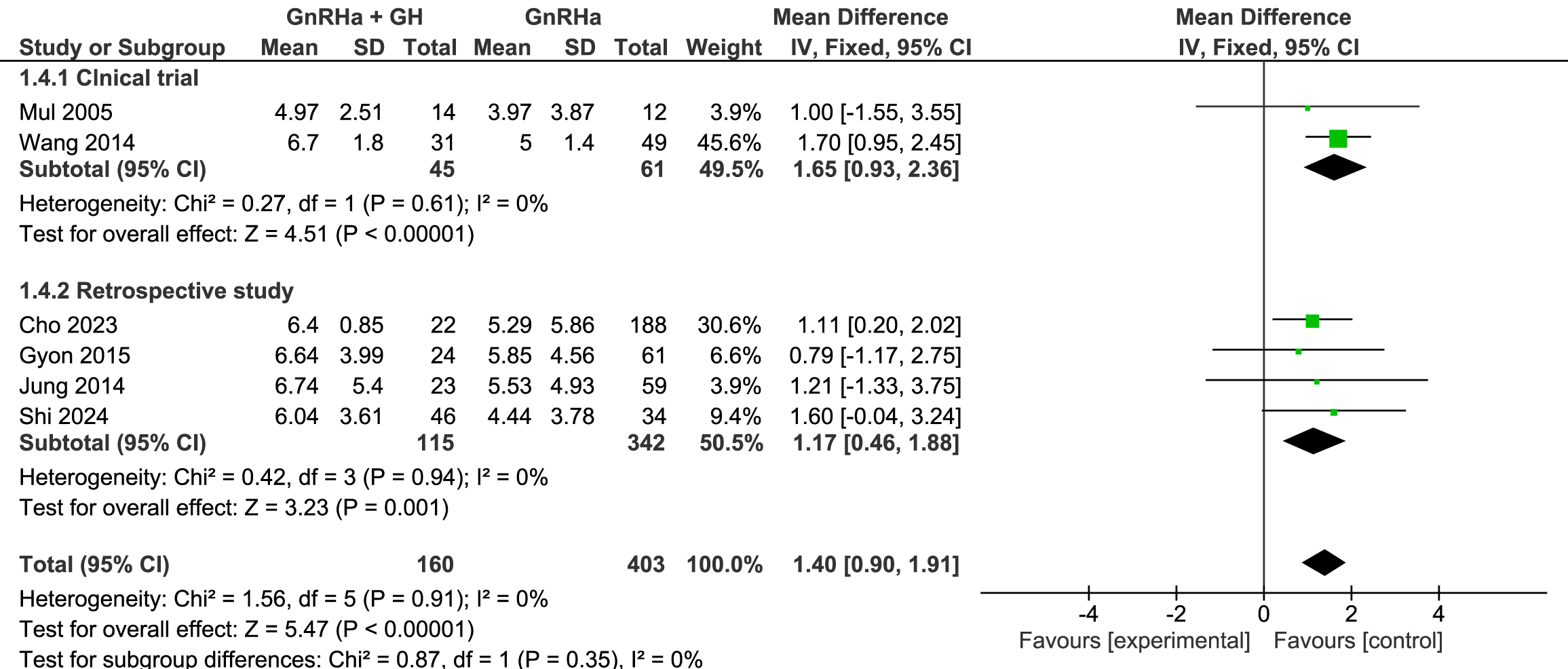

Supplement: Supplementary Figure 6 — Forest plot of subgroup analysis of growth velocity. [file Image6.tif]

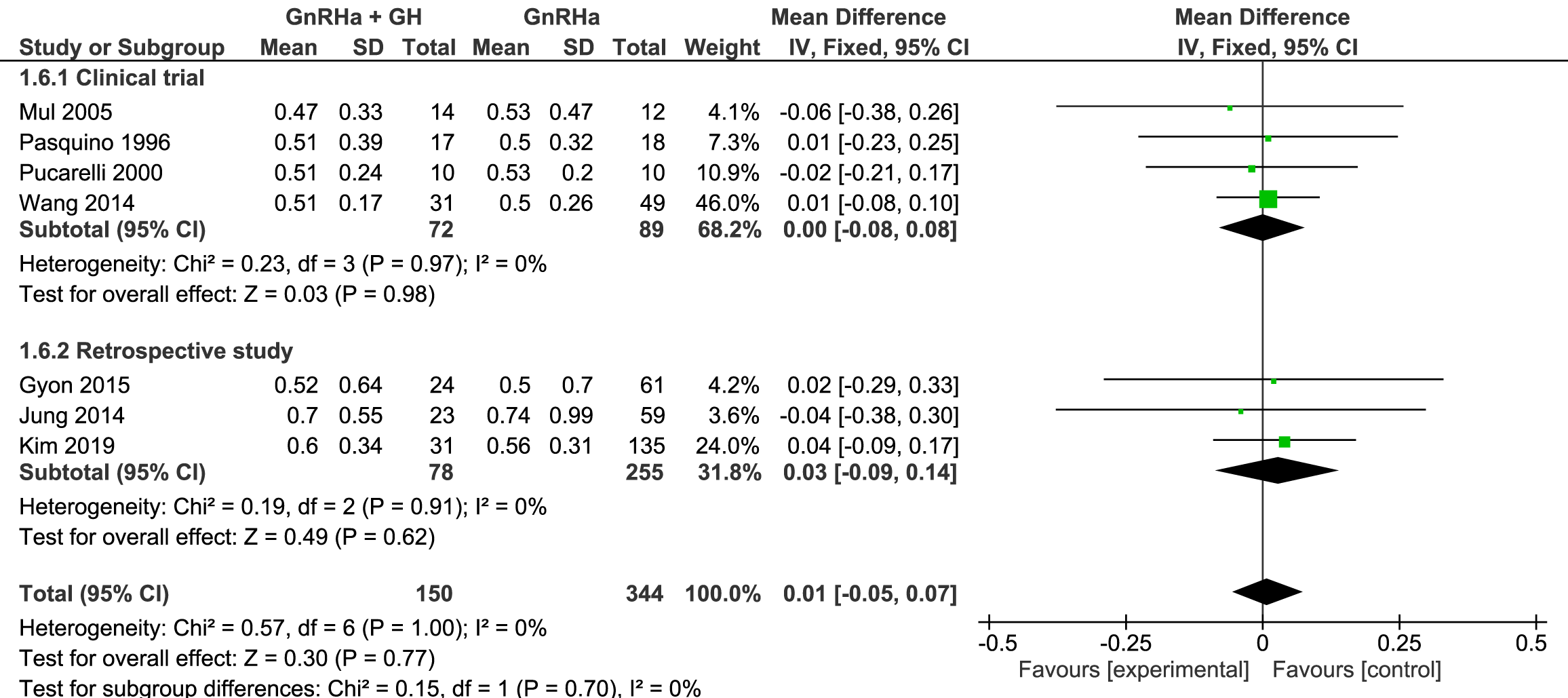

Supplement: Supplementary Figure 7 — Forest plot of subgroup analysis of bone maturation (ΔBA/ΔCA). [file Image7.tif]
